# Impact of switching to TAF/FTC/RPV, TAF/FTC/EVG/cobi and ABC/3TC/DTG on cardiovascular risk and lipid profile in people living with HIV: a retrospective cohort study

**DOI:** 10.1186/s12879-021-06304-3

**Published:** 2021-06-22

**Authors:** Andrea Giacomelli, Federico Conti, Laura Pezzati, Letizia Oreni, Anna Lisa Ridolfo, Valentina Morena, Cecilia Bonazzetti, Gabriele Pagani, Tiziana Formenti, Massimo Galli, Stefano Rusconi

**Affiliations:** 1 Department of Infectious Diseases, ASST Fatebenefratelli-Sacco, Luigi Sacco University Hospital, Milan, Italy; 2 III Infectious Diseases Unit, Luigi Sacco Department of Biomedical and Clinical Sciences DIBIC, University of Milan, Luigi Sacco Hospital, Legnano (MI), Italy; 3 Infectious Diseases Unit, Legnano General Hospital, ASST Ovest Milanese, Via G.B. Grassi 74, 20157 Milan, Italy

**Keywords:** Single tablet regimens, Tenofovir alafenamide, Framingham score, Cholesterol, Cardiovascular risk, Integrase inhibitors, Rilpivirine

## Abstract

**Background:**

We aimed to assess the overall cardiovascular and metabolic effect of the switch to three different single tablet regimens (STRs) [tenofovir alafenamide/emtricitabine/rilpivirine (TAF/FTC/RPV), TAF/FTC/elvitegravir/cobi (TAF/FTC/EVG/cobi) and ABC/lamivudine/dolutegravir (ABC/3TC/DTG)] in a cohort of people living with HIV/AIDS (PLWH) under effective ART.

**Methods:**

All PLWH aged above 18 years on antiretroviral treatment with an HIV-RNA < 50 cp/mL at the time of the switch to TAF/FTC/RPV, TAF/FTC/EVG/cobi and ABC/3TC/DTG were retrospectively included in the analysis. Framingham risk score modification after 12 months from the switch such as lipid profile and body weight modification were assessed. The change from baseline to 12 months in mean cardiovascular risk and body weight in each of the STR’s group were assessed by means of Wilcoxon signed-rank test whereas a mixed regression model was used to assess variation in lipid levels.

**Results:**

Five-hundred and sixty PLWH were switched to an STR regimen of whom 170 (30.4%) to TAF/FTC/EVG/cobi, 191 (34.1%) to TAF/FTC/RPV and 199 (35.5%) to ABC/3TC/DTG.

No difference in the Framingham cardiovascular risk score was observed after 12 months from the switch in each of the STR’s groups. No significant overtime variation in mean total cholesterol levels from baseline to 12 months was observed for PLWH switched to ABC/3TC/DTG [200 (SD 38) mg/dl vs 201 (SD 35) mg/dl; *p* = 0.610] whereas a significant increment was observed in PLWH switched to TAF/FTC/EVG/cobi [192 (SD 34) mg/dl vs 208 (SD 40) mg/dl; *p < 0.0001*] and TAF/FTC/RPV [187 (SD 34) mg/dl vs 195 (SD 35) mg/dl; *p = 0.027*]. In addition, a significant variation in the mean body weight from baseline to 12 months was observed in PLWH switched to TAF/FTC/EVG/cobi [72.2 (SD 13.5) kilograms vs 74.6 (SD 14.3) kilograms; *p < 0.0001*] and TAF/FTC/RPV [73.4 (SD 11.6) kilograms vs 75.6 (SD 11.8) kilograms; *p < 0.0001*] whereas no difference was observed in those switched to ABC/3TC/DTG [71.5 (SD 12.8) kilograms vs 72.1 (SD 12.6) kilograms; *p* = 0.478].

**Conclusion:**

No difference in the cardiovascular risk after 1 year from the switch to these STRs were observed. PLWH switched to TAF/FTC/EVG/cobi and TAF/FTC/RPV showed an increase in total cholesterol levels and body weight 12 months after the switch.

## Background

The progressive development of more convenient and effective antiretroviral regimens has turned an invariably fatal illness into a manageable chronic condition [1]. For several years the antiretroviral treatment (ART) was burdened by metabolic toxicities often leading to drug discontinuation and undermining the efficacy of ART [2–5].

The development and implementation of single tablet regimens (STRs) have been demonstrated to improve both adherence and convenience of drug uptake reducing the possible barriers to antiretroviral treatment related to pill burden or more than once a day antiretroviral administration [6–8]. The first STR combinations based on tenofovir disoproxil fumarate (TDF) although effective were potentially burdened by renal and bone toxicity especially when in combination with a drug requiring a boosting with ritonavir (RTV) or cobicistat (cobi) [9–11].

The advent of new STR combinations based on abacavir (ABC) or tenofovir alafenamide (TAF) helped to overcome this untoward drug effects [12–17]. Nevertheless, some concerns regarding the potential lipid and metabolic impact of TAF based regimens have been raised in recent years, in particular in those subjects switched from TDF based regimens [18, 19]. Moreover, the advent of integrase inhibitors (INIs) with an overall good tolerability profile have further improved the management of experienced HIV patients [17] with the possibility to overcome previous metabolic toxicity in particular by avoiding protease inhibitors (PIs) [20].

The overall cardiovascular risk modification after the switch to different STRs have not yet clearly elucidated. In the present study we aimed to assess the overall cardiovascular and metabolic effect of the switch to three different STRs [TAF/emtricitabine/rilpivirine (TAF/FTC/RPV), TAF/FTC/elvitegravir/cobi (TAF/FTC/EVG/cobi) and ABC/lamivudine/dolutegravir (ABC/3TC/DTG)] in a cohort of people living with HIV/AIDS (PLWH) under effective ART.

## Materials and methods

### Study design

Monocentric retrospective observational study.

### Setting

The study was conducted on the outpatient cohort of PLWH of the 3rd Infectious Diseases Division of Luigi Sacco Hospital, Milan, Italy, between December 2015 and June 2019.

### Participants

- All PLWH aged above 18 years on antiretroviral treatment with an HIV-RNA < 50 cp/mL at the time of the switch to TAF/FTC/RPV, TAF/FTC/EVG/cobi and ABC/3TC/DTG were included in the analysis excluding naïve patients and those with an HIV-RNA > 50 cp/mL at the time of the switch.

### Parameters

- Outcomes: cardiovascular risk assessed by Framingham risk score [21] at 12 months after the switch to one of the studied STRs (TAF/FTC/RPV, TAF/FTC/EVG/cobi and ABC/3TC/DTG). Total cholesterol, triglycerides, HDL cholesterol variation after 6 and 12 months from the switch. Body weight variation 12 months after the switch.
- Exposure: STRs (TAF/FTC/RPV, TAF/FTC/EVG/cobi and ABC/3TC/DTG)
- Predictors: we included in the analysis potential confounders such as age, gender, switch from TDF, PIs, INIs, RTV or cobi containing regimens, years on antiretroviral treatment, CD4 cell counts at the time of the switch, statin and other lipid lowering agents (fibrates, plant stanols and sterols, ezetimibe, cholestyramine and Omega-3 polyenic acids) exposure.

### Source of data and data handling

The data were extracted from an electronic database which included clinical and laboratory data. Al sensitive data have been anonymized and subsequently collected at the time of the switch and after 6 and 12 months from the switch:

- demographic and clinical data comprehensive of age, gender, HIV risk exposure.
- clinical data comprehensive of year of HIV diagnosis, antiretroviral history, years on antiretroviral treatment, AIDS diagnosis, HCV and/or HBV coinfection, CD4 cell count, CD4 cell count nadir, HIV-RNA determination, smoking habits, lipid lowering agents (statins, fibrate and other lipid lowering agents and time of lipid lowering agent start) and aspirin.
- Metabolic data comprehensive of Framingham score at the time of the switch and after 12 months, total cholesterol, triglycerides, HDL cholesterol and body weight.
- Reason leading to the switch of one of the investigated STRs.

### Potential Bias

Due to the retrospective nature of the study the analysis is exposed to selection and channelling bias. Moreover, it is possible that bias could be introduced by missing data. The monocentric design could limit the generalizability of our findings.

### Objectives

- The primary outcome was the assessment of Framingham risk score modification at 12 months after the switch in the different STRs.
- The secondary outcomes were the change at 12 months in body weight and the modification at 6 and 12 months of total cholesterol, triglycerides and HDL levels according to the different STR and the identification of potential predictors/effect modifiers.

### Statistical analysis

The study population is considered to be representative of the cohort of PLWH actually followed at our outpatient clinic.

- Sample size estimate: to evidence a between means (baseline and 12 months thereafter) minimum difference of at least 3.5% assuming a standard deviation of 11 in the cardiovascular risk assessed by Framingham risk score in each STR’s group (ABC/3TC/DTG, TAF/FTC/ECG/cobi and TAF/FTC/RPV) [22] it has been estimated a numerosity of 156 per-group patients to guarantee a power of 80% and a significance of α = 0.05.

- Statistical analysis: the data were identified and categorized according to be continous or categorical variables according to the objective of the study. A descriptive statistical analysis was performed and an inferential one assuming a significance level of α = 0.05.
- The analysis of cardiovascular risk was performed comparing the Framingham risk score at baseline and 12 months thereafter in each of the STR’s group by means of Wilcoxon signed-rank test.
- The dependent variable was for each analysis was: total cholesterol, triglycerides and body weight, respectively. Patients who introduced or modified a lipid lowering treatment during the study period were excluded.
- The variations within each STR’s group in total cholesterol, triglycerides and HDL cholesterol were evaluated trough a multivariable linear mixed effects regression model. The SAS PROC MIXED with random intercept was used to account for repeated measures. The intra individual variance was analysed through an autoregressive correlation of the first order.
- As covariates in the final model for lipid levels modification were included: age, gender, switch from TDF, PIs, INIs, baseline lipid levels, statin or lipid lowering agents’ exposure and adjusted for time effect after the switch.
- The analysis of body weight was performed comparing the baseline and 12 months’ values in each of the STR’s group by means of Wilcoxon signed-rank test.
- If the modification in body weight 12 months after the switch were found to be statistical significant a general linear model was built to assess factors associated to the overtime change in body weight. A logarithmic transformation with natural logarithm was applied to body weight (non-normally distributed variable).
- As covariates in the final model of body weight, and lipid levels modification were included: age, gender, switch from TDF, PIs, INIs, RTV or cobi, years on antiretroviral treatment, CD4 cell counts at the time of the switch.
- All of the statistical analyses were made using SAS software, version 9.4, and differences with *P* values of < 0.05 were considered statistical significant.

## Results

### Patients characteristics

During the study period 560 PLWH were switched to one of the investigated STRs: 170 (30.4%) to TAF/FTC/EVG/cobi, 191 (34.1%) to TAF/FTC/RPV, and 199 (35.5%) to ABC/3TC/DTG. Characteristics of patients at the time of the switch are reported in Table 1. PLWH enrolled were mainly males (77.5%) with a median age of 49 years [Inter Quartile Range (IQR) 41–55] and a median Body Mass Index (BMI) of 24.01 (IQR 21.97–25.98)]. Patients switched to ABC/3TC/DTG had a longer median antiretroviral history when compared to those treated with TAF/FTC/EVG/cobi and TAF/FTC/RPV (11.5 years vs 8.7 years vs 9.9 years, *p = 0.003*, respectively). Patients switched to ABC/3TC/DTG were more frequently switched from a PI-based regimens and less frequently from a regimen containing TDF when compared to those switched to TAF/FTC/EVG/cobi and TAF/FTC/RPV (41.6% vs 24.7% vs 6.8%, *p < 0.001* and 29.4% vs 77.6% vs 87.9%, *p < 0.001*; respectively).

**Table 1:** Characteristics of the cohort at the time of the switch to an STR

	Overall	TAF/FTC/EVG/cobi	TAF/FTC/RPV	ABC/3TC/DTG
	*N* = 560	*n* = 170 (30.4%)	*n* = 191 (34.1%)	*n* = 199 (35.5%)
**Gender, n (%)**				
**Female**	126 (22.5)	41 (24.1)	38 (19.9)	47 (23.6)
**Male**	434 (77.5)	129 (75.9)	153 (80.1)	152 (76.4)
**Age (years), median [IQR]**	49.00 [41.00, 55.00]	48.00 [38.25, 55.00]	48.00 [40.50, 54.00]	50.00 [43.00, 55.00]
**Risk group, n (%)**				
**Heterosexual**	233 (41.6)	75 (44.1)	76 (39.8)	82 (41.2)
**IVDU**	65 (11.6)	16 (9.4)	19 (9.9)	30 (15.1)
**MSM**	234 (41.8)	71 (41.8)	86 (45.0)	77 (38.7)
**Other**	28 (5.0)	8 (4.7)	10 (5.2)	10 (5.0)
**Smoke, n (%)**	220 (39.3)	77 (45.3)	65 (34.0)	78 (39.2)
**Weight (kg), median [IQR]**	72.00 [64.00, 80.00]	71.50 [63.05, 80.75]	73.00 [66.00, 81.00]	70.00 [63.00, 78.00]
**BMI, median [IQR]**	24.01 [21.97, 25.98]	23.70 [21.68, 25.86]	24.39 [22.49, 26.22]	23.94 [21.85, 25.97]
**Systolic blood pressure (mmHg), median [IQR]**	120.00 [110.00, 130.00]	120.00 [110.00, 130.00]	120.00 [110.00, 130.00]	120.00 [110.00, 130.00]
**Diastolic blood pressure (mmHg), median [IQR]**	80.00 [70.00, 80.00]	80.00 [70.00, 80.00]	80.00 [70.00, 80.00]	80.00 [70.00, 80.00]
**Previous therapy duration (years), median [IQR]**	10.36 [4.96, 16.88]	9.94 [3.19, 16.22]	8.71 [4.56, 15.11]	11.47 [6.70, 18.02]
**Previous AIDS, n (%)**	111 (19.8)	39 (22.9)	32 (16.8)	40 (20.1)
**Treated with ASA, n (%)**	38 (6.8)	9 (5.3)	10 (5.2)	19 (9.5)
**Treated with anti-hypertensive agent, n (%)**	101 (18.0)	31 (18.2)	32 (16.8)	38 (19.1)
**Treated with oral hypoglycemic agents / insulin, n (%)**	18 (3.2)	3 (1.8)	8 (4.2)	7 (3.5)
**Treated with lipid-lowering agents other than statins** ^**a**^**, n (%)**	43 (7.7)	16 (9.4)	12 (6.3)	15 (7.5)
**Treated with statins, n (%)**	88 (15.7)	27 (15.9)	33 (17.3)	28 (14.1)
**HCV, n (%)**	121 (21.6)	28 (16.5)	40 (20.9)	53 (26.6)
**HBV, n (%)**	25 (4.5)	14 (8.2)	6 (3.1)	5 (2.5)
**Switch from PI, n (%)**	137 (24.6)	42 (24.7)	13 (6.8)	82 (41.6)
**Switch from TDF, n (%)**	357 (64.1)	132 (77.6)	167 (87.9)	58 (29.4)
**Switch from RTV / cobi, n (%)**	193 (34.5)	113 (66.5)	16 (8.4)	64 (32.2)
**Switch from INI, n (%)**	143 (25.5)	97 (57.1)	5 (2.6)	41 (20.6)
**CD4+ (cells/mmc), median [IQR]**	710.50 [560.00, 893.00]	681.00 [535.00, 878.75]	711.00 [566.50, 862.00]	744.00 [580.00, 961.00]
**Creatinine (mg/dl), median [IQR]**	0.90 [0.79, 1.03]	0.90 [0.79, 1.01]	0.94 [0.80, 1.04]	0.89 [0.76, 1.04]
**eGFR ml/min, median [IQR]**	98.49 [79.96, 118.06]	97.31 [80.67, 118.45]	101.24 [81.70, 115.49]	96.92 [75.87, 118.96]
**Triglycerides (mg/dl), median [IQR]**	115.00 [87.00, 166.00]	110.50 [88.50, 169.25]	110.50 [81.25, 150.25]	122.00 [91.00, 173.00]
**Total cholesterol (mg/dl), median [IQR]**	193.00 [171.00, 215.00]	192.50 [173.75, 213.50]	186.00 [169.00, 209.00]	200.00 [174.00, 224.00]
**HDL cholesterol (mg/dl), median [IQR]**	43.00 [37.00, 52.00]	43.00 [35.50, 54.00]	43.00 [37.00, 49.50]	44.00 [36.00, 52.00]
**LDL cholesterol (mg/dl), median [IQR]**	120.30 [101.40, 143.05]	121.80 [102.00, 140.80]	115.80 [100.75, 139.50]	121.20 [102.40, 153.90]
**Framingham risk score, median [IQR]**	8.25 [4.19, 16.03]	9.18 [3.92, 18.02]	7.23 [3.47, 14.11]	9.23 [5.13, 15.99]

^a^fibrates, plant stanols and sterols, ezetimibe, cholestyramine and Omega-3 polyenic acids

*List of abbreviations*: *n* number, *IQR* Inter Quartile Range, *MSM* men who have sex with men, *IVDU* intravenous drug users, *PI* protease inhibitors, *INI* integrase inhibitors, *TDF* tenofovir disoproxil fumarate, *HDL* high density lipoprotein, *LDL* low density lipoprotein, *CD* cluster of differentiation, *RTV* ritonavir, *cobi* cobicistat, *eGFR* estimated glomerular filtration rate, *BMI* body mass index, *ASA* acetil salicylic acid, *AIDS* acquired immune deficiency syndrome, *HCV* hepatitis C virus, *HBV* hepatitis B virus, *TAF* tenofovir alafenamide, *FTC* emtricitabine, *3TC* lamivudine, *ABC* abacavir, *DTG* dolutegravir, *RPV* rilpivirine

### Reasons of switch

The main reason of switch toward an STR was related to simplification driven by concomitant comorbidities [323 (57.6%) patients] followed by simplification to improve adherence [155 (27.7%) patients], other reasons [62 (11.1%) patients] and drug-drug interactions [20 (3.6%) patients].

### Cardiovascular risk assessment

**Figure 1:**
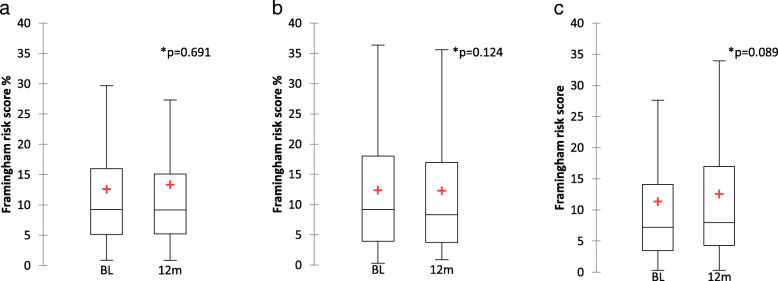
Fig. 1 **a**, boxplots of Framingham risk values (%) at baseline and after 12 months from the switch to ABC/3TC/DTG. **b**, boxplots of Framingham risk values at baseline and after 12 months from the switch to TAF/FTC/EVG/cobi. **c**, boxplots of Framingham risk values at baseline and after 12 months from the switch to TAF/FTC/RPV. The cross indicates the mean value. List of abbreviations: BL, baseline; m, months. *Wilcoxon signed rank test

No difference in the Framingham cardiovascular risk score was observed after 12 months from the switch in each of the STR’s groups (Fig. 1). In particular, mean Framingham risk score was 12.6% [Standard Deviation (SD) 11.5] at baseline and 13.4% (SD 14.1) after 12 months from the switch to ABC/3TC/DTG (*p* = 0.691); 12.4% [Standard Deviation (SD) 11] and 12.3% (SD 11.1) from the switch to TAF/FTC/EVG/cobi (*p* = 0.124); 11.3% [Standard Deviation (SD) 11.9] and 12.6% (SD 12.5) from the switch to TAF/FTC/RPV (*p* = 0.089), respectively.

### Serum lipid profile

**Figure 2:**
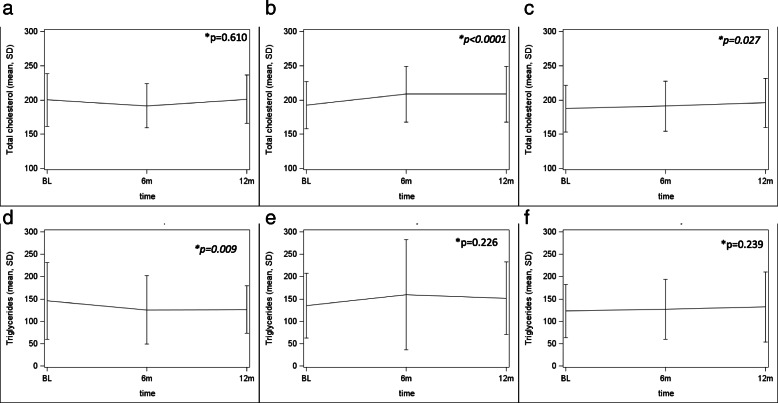
Fig. 2 **a**, mean total cholesterol variation (mg/dL) after the switch to ABC/3TC/DTG. **b**, mean total cholesterol variation (mg/dL) after the switch to TAF/FTC/EVG/cobi. **c**, mean total cholesterol variation (mg/dL) after the switch to TAF/FTC/RPV. **d**, mean triglycerides variation (mg/dL) after the switch to ABC/3TC/DTG. **e**, mean triglycerides variation (mg/dL) after the switch to TAF/FTC/EVG/cobi. **f**, mean triglycerides variation (mg/dL) after the switch to TAF/FTC/RPV. List of abbreviations: BL, baseline; m, months; SD, Standard Deviation. *Mixed linear regression models comparing mean values at baseline (switch), 6 and 12 months thereafter

Mean total cholesterol and triglycerides levels at the time of the switch and at 6 and 12 months thereafter in the investigated STR’s groups are reported in Fig. 2.

No significant overtime variation in mean total cholesterol levels from baseline to 12 months was observed for PLWH switched to ABC/3TC/DTG [200 (SD 38) mg/dl vs 201 (SD 35) mg/dl; *p* = 0.610] whereas a significant increment was observed in PLWH switched to TAF/FTC/EVG/cobi [192 (SD 34) mg/dl vs 208 (SD 40) mg/dl; *p < 0.0001*] and TAF/FTC/RPV [187 (SD 34) mg/dl vs 195 (SD 35) mg/dl; *p = 0.027*].

A significant reduction in mean triglycerides levels was observed for PLWH switched to ABC/3TC/DTG from baseline to 12 months [145 (SD 85) mg/dl vs 126 (SD 52) mg/dl; *p = 0.009*] whereas no differences were observed in those switching to TAF/FTC/EVG/cobi [135 (SD 72) mg/dl vs 151 (SD 81) mg/dl; *p* = 0.226] and TAF/FTC/RPV [123 (SD 59) mg/dl vs 132 (SD 78) mg/dl; *p* = 0.239].

A significant slight increase in mean HDL levels from baseline to 12 months were observed in all the STR’s groups [ABC/3TC/DTG: 46 (SD 14) mg/dl vs 48 (SD 12) mg/dl; *p = 0.040;* TAF/FTC/EVG/cobi: 45 (SD 13) mg/dl vs 46 (SD 11) mg/dl; *p = 0.045*; TAF/FTC/RPV: 44 (SD 12) mg/dl vs 47 (SD 11) mg/dl; *p = 0.011*].

In the multivariable model a correlation between female gender [estimate 16.4 mg/dL, Standard error (SE) 5.7; *p < 0.005*] and statin exposure [estimate − 22.8 mg/dL, SE 7.4; *p = 0.002*] was observed for total cholesterol modification in PLWH switched to TAF/FTC/EVG/cobi, whereas switching from a TDF containing regimen [estimate 19.4 mg/dL, SE 6.7; *p = 0.005*] and statin exposure [estimate − 15.9 mg/dL, SE 6.2; *p = 0.012*] resulted independently associated to total cholesterol modification in those switching to TAF/FTC/RPV.

The switch from TDF containing regimens (estimate 29.2 mg/dL, SE 8.5; *p = 0.0007*) resulted independently associated to triglycerides modifications in PLWH switched to ABC/3TC/DTG.

### Body weight

**Figure 3:**
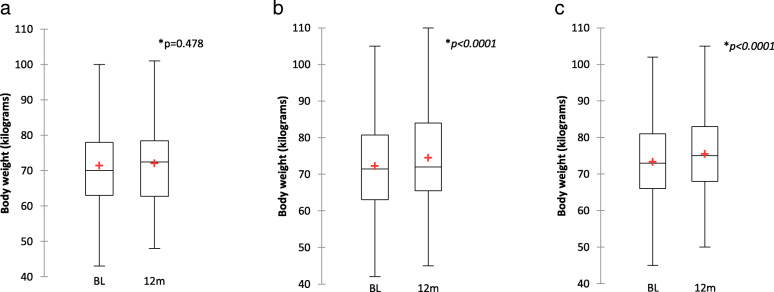
Fig. 3 **a**, boxplots of body weight values (kilograms) at baseline and after 12 months from the switch to ABC/3TC/DTG. **b**, boxplots of body weight values at baseline and after 12 months from the switch to TAF/FTC/EVG/cobi. **c**, boxplots of body weight values at baseline and after 12 months from theswitch to TAF/FTC/RPV. The cross indicates the mean value. List of abbreviations: BL, baseline; m, months. baseline; m, months. *Wilcoxon signed rank test

The comparison of body weight at the time of the switch to one of the investigated STRs and after 12 months are depicted in Fig. 3. A significant variation in the mean body weight from baseline to 12 months was observed in PLWH switched to TAF/FTC/EVG/cobi [72.2 (SD 13.5) kilograms vs 74.6 (SD 14.3) kilograms; *p < 0.0001*] and TAF/FTC/RPV [73.4 (SD 11.6) kilograms vs 75.6 (SD 11.8) kilograms; *p < 0.0001*] whereas no statistical significant difference was observed in those switched to ABC/3TC/DTG [71.5 (SD 12.8) kilograms vs 72.1 (SD 12.6) kilograms; *p* = 0.478].

In the general linear model restricted to PLWH switched to TAF/FTC/EVG/cobi an association between body weight variation at 12 months and male gender (estimate − 0.04 log, SE 0.01; *p < 0.001*), CD4 cell count (per 200 more) (estimate − 0.006 log, SE 0.003; *p < 0.0001*), the switch from a TDF containing regimen (estimate 0.063 log, SE 0.013; *p < 0.0001*) and the switch from an INI containing regimens (estimate − 0.064 log, SE 0.13; *p < 0.0001*) was observed.

In the general linear model restricted to PLWH switched to TAF/FTC/RPV an association between body weight variation at 12 months and the switch from a PI containing regimen (estimate − 0.137 log, SE 0.039; *p = 0.001*) and the switch from an RTV/cobi containing regimen (estimate 0.089 log, SE 0.035; *p = 0.013*) was observed.

### Lipid lowering agents and aspirin prescription

In subjects with a baseline Framingham cardiovascular risk score above 10, 25.3% PLWH were under statin, 12.4% received another lipid lowering agent and 14.1% were treated with aspirin.

A slightly higher percentage was observed in subjects with a Framingham score above 20%: 13.7% under statin, 28.8% treated with another lipid lowering drug and 20.5% treated with aspirin.

## Discussion

In the present study we observed no difference in the cardiovascular risk assessed by Framingham score after 12 months from the switch to TAF/FTC/EVG/cobi, TAF/FTC/RPV and ABC/3TC/DTG. Nevertheless, PLWH switched to TAF/FTC/EVG/cobi and TAF/FTC/RPV showed an increase in total cholesterol levels and body weight 12 months after the switch.

In our cohort of PLWH under effective ART, we observed that the treatment modifications were mainly driven by a proactive switch in the presence of comorbidities or to reduce the pill burden in order to improve adherence [6–8]. If on the one hand the switch to one STR is able to improve the adherence and treatment convenience [6, 23], on the other it is important to assess the overall cardiovascular risk before the treatment simplification through one of the validated tools recommended by international guidelines [24, 25].

In our cohort we observed no difference in the Framingham risk score after 12 months from the switch to one of the different STRs studied suggesting that ART modification has little effect on the overall cardiovascular risk [15, 26] and consequently it is unlikely to obtain significant modification in cardiovascular risk with the only modification of the ART regimen.

Nevertheless, the changes in lipid profile and body weigh observed after the switch to TAF/FTC/EVG/cobi and TAF/FTC/RPV warrant further discussion.

In particular, it was observed that both the switch to TAF/FTC/EVG/cobi and TAF/FTC/RPV were burdened by a slight increase in total cholesterol level. This observation could be partially explained by the high proportion of PLWH switching from a TDF containing regimen in both groups (77.6 and 87.9%, respectively) and this association was confirmed to be independent in the multivariable model restricted to patients switched to TAF/FTC/RPV. This finding is expected considering the possible TDF statin-like effect [19] with an increase in particular of total cholesterol after TDF interruption [18]. Our observation of an increase in total cholesterol levels after the switch to TAF/FTC/EVG/cobi are in line with the report by Kuo et al. showing an increase in lipid level associated with weight gain in virologically suppressed PLWH switched to TAF/FTC/EVG/cobi [27]. In particular, the potential role of different ART regimens in affecting weight gain has been investigated in recent years [28–31]. In our cohort we observed that the switch to TAF/FTC/EVG/cobi and TAF/FTC/RPV were both burdened by a significant increase in body weigh 12 months after the switch. The explanation to this observation could be partially found in the association reported in observational studies with INIs [28, 30, 31] and TAF [32] exposure.

In a recent conference presentation Mallon and co-workers showed an independent effect of a switch from TDF to TAF on weight gain irrespectively of being concomitantly switched to an INI-containing regimen [33]. In addition, Surial et al evidenced in the Swiss HIV Cohort how the replacement of TDF with TAF was associated with adverse metabolic changes, including weight gain, development of obesity, and worsening serum lipid levels comprehending total cholesterol, high-density lipoprotein cholesterol, low-density lipoprotein cholesterol and triglyceride [34]. The potential weight gain associated with ART should always be discussed with the patient and a counselling regarding dietary and physical activity should be provided.

A low prescription of statin such as of aspirin was observed in our cohort. This finding is in line with previous reports of low statin use in PLWH mainly caused by a combination of fear to drug-drug interaction and unproven expected higher incidence of adverse events in PLWH [35–37]. This finding is overall worrisome and an effort should be made to fill this gap in the prevention of cardiovascular events [38]. In parallel a low aspirin prescription (20.5%) in subjects with a 10 year Framingham risk above 20% suggesting that although the current literature evidence in support aspirin prescription to reduce cardiovascular risk mortality still remain an unmet need [39].

### Study limitations

Our study has several limitations. First, the retrospective design could have introduced bias into the analysis related to missed data. Second, the choice of the switch was based on clinical judgement and, although the demographic characteristics of the three group were not significantly different, some unbalancing between groups were present and this makes the between groups comparisons unfeasible. Third, the Framingham risk score does not take into account the potential role of ABC as a potential independent additive risk factor for cardiovascular risk. Fourth, considering the observational nature of our study no causality link could be inferred between the switch to one of the different STRs and the investigated outcomes but only associations that should be carefully interpreted in light of potential residual confounders that have not been taken into account into the analysis. In the end, the monocentric design limits the generalizability of our findings to other settings. Nevertheless, the study population included in the present study is overall representative of PLWH attending our HIV outpatient clinic.

## Conclusions

No difference in the cardiovascular risk after 1 year from the switch to ABC/3TC/DTG, TAF/FTC/EVG/cobi and TAF/FTC/RPV were observed. PLWH switched to TAF/FTC/EVG/cobi and TAF/FTC/RPV showed an increase in total cholesterol levels and body weight 12 months after the switch although the overall impact on the cardiovascular risk assessed by Framingham risk score seems to be limited. An important gap in preventing cardiovascular risk in a high-risk population by means of pharmacological interventions (i.e. lipid lowering agents and aspirin) was found. These observations highlight the need of proactive intervention to reduce the long term cardiovascular risk in the same way we are proactive in simplify ART regimens.

## Data Availability

The dataset will be made available upon reasonable request. Andrea Giacomelli and Stefano Rusconi should be contacted if someone wants to request the data from this study.

## Abbreviations

STR: Single tablet regimens
TAF/FTC/RPV: Tenofovir alafenamide/emtricitabine/rilpivirine
TAF/FTC/EVG/cobi: Tenofovir alafenamide/emtricitabine/elvitegravir/cobicistat
ABC/3TC/DTG: Abacavir/lamivudine/dolutegravir
IQR: Inter Quartile Range
CI: Confidence Interval
PLWH: People living with HIV AIDS
SE: Standard error
ART: Antiretroviral treatment
TDF: Tenofovir disoproxil fumarate
RTV: Ritonavir
PIs: Protease inhibitors
